# Metabolic syndrome and its components among women with polycystic ovary syndrome: a systematic review and meta-analysis

**DOI:** 10.15171/jcvtr.2018.10

**Published:** 2018-05-28

**Authors:** Jamal Hallajzadeh, Maliheh Khoramdad, Nahid Karamzad, Amir Almasi-Hashiani, Ali Janati, Erfan Ayubi, Reza Pakzad, Mark J.M. Sullman, Saeid Safiri

**Affiliations:** ^1^Managerial Epidemiology Research Center, Department of Public Health, School of Nursing and Midwifery, Maragheh University of Medical Sciences, Maragheh, Iran; ^2^Department of Epidemiology and Biostatistics, Faculty of Health, Kermanshah University of Medical Sciences, Kermanshah, Iran; ^3^Nutrition Research Center, Department of Biochemistry and Diet Therapy, School of Nutrition and Food Sciences, Tabriz University of Medical Sciences, Tabriz, Iran; ^4^Department of Epidemiology and Reproductive Health, Reproductive Epidemiology Research Center, Royan Institute for Reproductive Biomedicine, ACECR, Tehran, Iran; ^5^Iranian Center of Excellence in Health Management, Department of Health Services Management, School of Management and Medical Informatics, Tabriz University of Medical Sciences, Tabriz, Iran; ^6^Department of Epidemiology, School of Public Health, Shahid Beheshti University of Medical Sciences, Tehran, Iran; ^7^Department of Epidemiology, Faculty of Health, Ilam University of Medical Sciences, Ilam, Iran; ^8^Middle East Technical University, Northern Cyprus Campus, Güzelyurt/Morphou, Northern Cyprus; ^9^Department of Epidemiology and Biostatistics, School of Public Health, Tehran University of Medical Sciences, Tehran, Iran

**Keywords:** Global, Metabolic Syndrome, Prevalence, Polycystic Ovary Syndrome, Meta-Analysis

## Abstract

***Introduction:*** The objectives of this study were to provide an estimate of the prevalence of metabolic syndrome (MetS ) and its components among women with PCOS; and calculate the odds ratio (OR) for MetS (using different definitions of MetS) in women with PCOS, compared to healthy controls.

***Methods:*** All of the relevant databases were used to search for appropriate articles that were published during the period 2003-2016. We included observational studies (cross-sectional, comparative cross-sectional) among women who met the inclusion criteria. The random-effect models were used to pool the prevalence of MetS and its components among PCOS women. This model was also applied to the pooled OR assessing the association between MetS and PCOS.

***Results:*** The pooled prevalence of MetS among PCOS women was found to be 26.30% (95% CI: 23.68–28.93), but varied from 7.10% (95% CI: 1.64-12.56) to 37.50% (95% CI: 28.84-46.16), depending upon the diagnostic criteria used. Low high-density lipoprotein cholesterol (HDL) - 61.87% (95% CI: 53.31–70.43) and high waist circumference (WC)- 52.23% (95% CI: 43.84–60.61) were the most common components of MetS in PCOS women. Compared to healthy controls, the overall pooled (OR) of MetS in PCOS patients was 2.09 (95% CI: 1.67-2.60), but this ranged from 0.31 (95% CI: 0.13-0.74) to 4.69 (95% CI: 2.09-10.52), depending upon the diagnostic criteria used.

***Conclusion:*** Women with PCOS had a much higher prevalence of MetS than was found among the healthy controls. Furthermore, as low HDL and high WC were the most common components of MetS in PCOS women, these two components specifically need to be addressed in prevention strategies.

## Introduction

Polycystic ovary syndrome (PCOS) is one of the most important clinical and public health problems facing women, and has been reported to affect more than 20% of reproductive aged women.^1^ It has been estimated that the total cost of evaluating and providing care for reproductive-aged PCOS women in the United States is around $4.36 billion, excluding any potential obstetric complications.^2^ This syndrome has been found to be associated with significant adverse sequelae that can degrade long-term health and well-being. The short-term morbidities of PCOS include dermatologic, reproductive, and mood disturbances, while the longer-term morbidities of PCOS include vascular dysfunction, neoplastic, and mental health disorders.^3^ In addition, women with PCOS tend to have other abnormalities, such as: hypertension, dyslipidemia, insulin resistance, impaired glucose tolerance, obesity, and diabetes mellitus. Consequently, women with PCOS are highly susceptible to metabolic syndrome (MetS).^4,5^ MetS has recently been estimated to have a prevalence of about 23.8%-53.3% among women suffering from PCOS.^6-8^ A similar range in the prevalence of MetS components has also been reported.^8-10^ The variation in research findings may be due to differences among the participants included in each study, such as: diet, lifestyle and genetic factors. However, one obvious reason for the wide range in the prevalence of MetS is the definition used to diagnose MetS.^11^ There are a number of definitions of MetS, which mainly differ according to the number of components required and the cut-off points used (Table 1).

**Table 1:** Summary of the MetS definitions

**Definitions**	**WHO**	**NCEP-ATP III**	**IDF**	**EGIR**	**AACE**	**AHA/NHLBI**	**ATP III**	**JS 2009**
Number of Criteria	Two or more of:	Three or more of:	Two or more of :	Two or more of:	Obesity and two or more of:	Three or more of:	Three or more of:	Three or more of:
Obesity	BMI > 30 and/or WHR > 0.9 (men), WHR > 0.85 (women)	WC ≥ 102 cm (men), WC ≥ 88 cm (women	WC ≥ 94 cm men, WC ≥ 80 cm women	WC ≥ 94 cm (men, WC ≥80 cm (women)	WC ≥ 102 cm (men), WC ≥ 88 cm (women	BMI ≥ 30 kg/m2	WC ≥ 102 cm (men), WC ≥ 88 cm (women	Population- and country-specific definitions
Blood pressure mm Hg	≥ 140/90	≥ 130/85 or treatment	≥130/≥85 or treatment	≥ 140/90	≥ 130/85 or treatment	≥130/85 mm Hg or previous hypertension diagnosis	≥ 130/85 or treatment	≥ 130/85 or treatment
Dyslipidmia
HDL-C	≥ 35 mg/dL (0.9 mmol/L) in men or ≥ 39 mg/dL (≥ 1.0 mmol/L) in women	≥ 40 mg/dL (1.03 mol/L) in men, ≥ 50 mg/dL (1.29 mmol/L) in women, or treatment	≥ 40 mg/dL (1.03 mol/L) in men, ≥ 50 mg/dL (1.29 mmol/L) in women, or treatment	≥ 39 mg/dL (1.0 mmol/L) or treatment	≥ 40 mg/dL (1.03 mol/L) in men, ≥ 50 mg/dL (1.29 mmol/L) in women, or treatment	≥ 40 mg/dL (1.03 mol/L) in men, ≥ 50 mg/dL (1.29 mmol/L) in women	≥ 40 mg/dL (1.03 mol/L) in men, ≥ 50 mg/dL (1.29 mmol/L) in women	≥ 40 mg/dL (1.03 mol/L) in men, ≥ 50 mg/dL (1.29 mmol/L) in women, or treatment
Triglycerides	≥178 mg/dL(2.0 mmol/L) or treatment	≥150 mg/dL (1.7 mmol/L) or treatment	≥150 mg/dL (1.7 mmol/L) or treatment	≥150 mg/dL (1.7 mmol/L)	≥150 mg/dL (1.7 mmol/L) or treatment	≥150 mg/dL (1.7 mmol/L) or treatment	≥150 mg/dL (1.7 mmol/L)	≥150 mg/dL (1.7 mmol/L) or treatment
Glucose Intolerance or Fasting Plasma Glucose	≥110 mg/dL (6.1 mmol/l), DM, IGT, IR	≥100 mg/dL (5.6 mmol/L) or T2D	≥100 mg/dL (5.6 mmol/L) or T2D	≥110 mg/dL (6.1 mmol/L)	≥110 mg/dL (6.1 mmol/l), or treatment	≥100 mg/dL (5.6 mmol/L) or T2D	≥110 mg/dL (6.1 mmol/L)	≥100 mg/dL (5.6 mmol/L) or T2D

BMI = body mass index; JC= Joint Consensus; DM = diabetes mellitus; EGIR = European Group against Insulin Resistance; HDL-C = high-density lipoprotein cholesterol; IDF = International Diabetes Federation; IGT = impaired glucose tolerance; IR = insulin resistance; NCEP ATPIII = National Cholesterol Education Program Adult Treatment Panel; AACE= American Association of Clinical Endocrinologists; AHA/NHLBI= The American Heart Association / National Heart, Lung, and Blood Institute; JS= Joint Statement; T2 D, type II diabetes mellitus; WC = waist circumference; WHO = World Health Organization; WHR = waist hip ratio.

The relationship between PCOS and MetS has been studied a number of times, with several studies reporting MetS to be more prevalent among women with PCOS than among women of the same age without PCOS.^11-13^ In contrast, there are also studies which have reported a higher rate of MetS among healthy women, than among those diagnosed with PCOS.^14^

In fact, despite a number of studies being conducted in different parts of the world, there has not yet been a comprehensive study of the prevalence of MetS and its components among women diagnosed with PCOS^15^ Therefore, the objectives of this systematic review and meta-analysis were to: 1) update the prevalence of MetS in women with PCOS, based on commonly used definitions of MetS; 2) determine the prevalence of MetS components among this group of women; and 3) calculate the odds ratio for MetS (using different definitions of MetS) among women with PCOS, in comparison to healthy controls.

## Methods

### Search strategy and study selection

We conducted a systematic review and meta-analysis using the “Preferred Reporting Items for Systematic Reviews and Meta-Analysis” (PRISMA) guidelines.^16^ The review included all articles published from 2003 to 2016 which measured the prevalence of MetS and/or the components of this syndrome (i.e., waist circumference - WC, blood pressure - BP, high-density lipoprotein cholesterol -HDL-C, triglycerides - TG, fasting blood sugar - FBS) among women diagnosed with PCOS.

The keywords were initially extracted using the medical subject headings (MESH) in Medline, which were: “metabolic syndrome”, “dysmetabolic syndrome”, “cardiovascular syndrome”, “insulin resistance syndrome”, “polycystic ovary syndrome”, “PCOS”, “Prevalence”, “odds ratio”, “cross-sectional studies”, “comparative cross-sectional studies” and “case-control studies”. These keywords were then used to search the following databases: PubMed, Web of Science, Medline, Scopus, Embase, CABI, CINAHL, DOAJ, and Index Medicus for Eastern Mediterranean Region-IMEMR. In addition, Google Scholar was used to search the grey literature, as recommended by previous research,^17^ using the abovementioned search strategy. Finally, in an attempt to gather additional articles, an expert in the subject area was consulted.

### Inclusion and exclusion criteria

Articles were included from observational studies (cross-sectional, and comparative cross-sectional) which met the following criteria: (a) diagnosis of PCOS using the Rotterdam criteria, which includes women with at least two of the three symptoms (i.e., polycystic ovaries, oligo-ovulation or anovulation and clinical and/or biochemical signs of hyperandrogenism); and (b) MetS, or its components, diagnosed in women with PCOS (or those without PCOS), based upon a standard definition. Original articles were included irrespective of age or race. However, studies were excluded where: (a) an unclear definition of MetS was provided; (b) the data reported were insufficient to determine the prevalence of MetS; and (c) patients were suffering from other clinical disorders. In cases where the data were not included in the published articles, we contacted one of the authors (first/corresponding authors) at least twice in an attempt to obtain the required information.

### Data extraction and quality assessment

Two researchers (SS and JH), independently took responsibility for entering data in the review and a third researcher was consulted when any differences were identified. The variables extracted from the articles were: study characteristic (first author’s name, date of publication, and country of origin); participant characteristics (gender, age, and sample size); MetS prevalence, as well as the definition(s) used; and the prevalence of MetS components (WC, BP, HDL-C, TG, FBS). The quality of the study was also measured using the STROBE checklist (22 items). Those which met the minimum acceptable quality criteria (>15 items) were included in the analysis.^18^ It is important to mention that the risk was assessed using the Newcastle–Ottawa scale.^19^ The research design, recruitment strategy, response rate, representativeness of the sample, objectivity of the outcome, power calculation provided, and appropriate statistical analyses were also evaluated.^19^ The minimum and maximum scores were 0 and 9, respectively. The studies were then categorized as low risk (≥6), moderate risk (<6 but >3) and high risk (<3).

### Statistical analysis

A random-effects model was used to analyze the prevalence of MetS, and its components, among women with PCOS. An odds ratio (OR) was used to illustrate the association between MetS and PCOS, also using a random effects model. Heterogeneity between studies was examined using the I^2^ index and a random-effects model was again used where heterogeneity was identified (I^2^> 0.6). Meta-regression was used to identify the source of heterogeneity and publication bias was investigated using a funnel plot and Egger’s test.^20^ All statistical tests were conducted using Review Manager (RevMan) version 5.3. (Copenhagen: The Nordic Cochrane Centre, The Cochrane Collaboration, 2014) and Stata software version 13 (Stata Corp, College Station, TX, USA).

## Results

**Figure 1:**
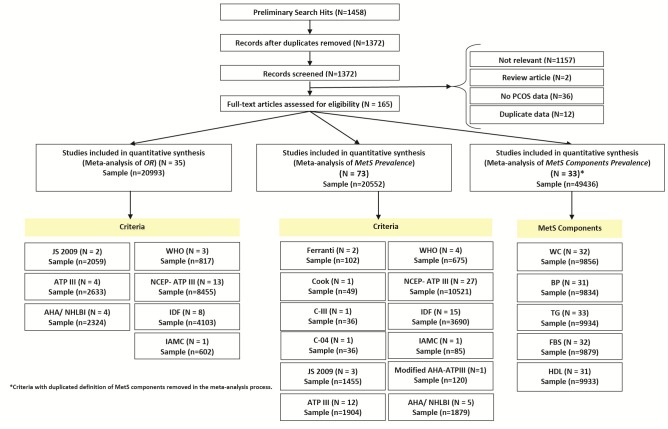
Flow diagram of the study selection process.

A total of 1458 records were identified through the combined search of the databases. However, following elimination of the non-eligible studies, a total of 72 cross-sectional studies and 35 comparative cross-sectional studies were retained to estimate the prevalence and risk of MetS among women with PCOS. Figure 1 depicts the flow chart used in the study selection. Articles were identified from 20 different countries during the period 2003-2015, with the majority of these originating from the United States. Surprisingly, there were no articles identified from Africa. The age range of individuals who had taken part in these studies ranged from 15-54 years old. The MetS definition used in this study was based on the NCEP-ATP III criteria. The characteristics of the studies included in this research are reported in Tables 2 and 3.

**Table 2:** Prevalence of metabolic syndrome in women with and without PCOS

**First Author**	**Country**	**Criteria**	**Study year**	**Publication Year**	**Characteristics of People with PCOS**				**Characteristics of People without PCOS**				**Reference**
					**Age Range**	**Mean Age**	**Total Sample**	**Prev. MetS**	**Age Range**	**Mean Age**	**Total Sample**	**Prev. MetS**	
Kyrkou G	Greece	IDF	-	2015	-	24.7	230	12.6	-	24.1	155	1.9	^21^
Romanowski MD	Brazil	NCEP-ATP III	2008-2009	2015	-	26.8	101	32.7	-	33.7	77	19.5	^22^
Romanowski MD	Brazil	IDF	2008-2009	2015	-	26.8	101	44.6	-	33.7	77	28.6	^22^
Pillai BP	India	IDF	2010-2012	2015	12-41	24.8	121	52.0	-	-	-	-	^23^
Pillai BP	India	NCEP-ATP III	2010-2012	2015	12-41	24.8	121	30.6	-	-	-	-	^23^
Madani T	Iran	NCEP-ATP III		2015	-	28.6	624	19.7	-	-	-	-	^24^
Shabir I	India	IDF	2009-2010	2014	13-28	23.0	37	27.0	-	-	-	-	^25^
Shabir I	India	ATP III	2009-2010	2014	13-28	23.0	37	22.0	-	-	-	-	^25^
Figurova J	Slovakia	NCEP-ATP III	2010-2013	2014	20-41	29.2	99	21.2	-	-	-	-	^26^
Tehrani FR	Iran	IAMC	2010-2012	2014	18-45	29.1	85	7.1	-	33.9	517	19.53	^27^
Rong Li	China	NCEP-ATP III	-	2014	19-45	29.1	833	19.1	-	32.3	2732	14.7	^28^
Kim MJ	Korea	NCEP-ATP III	2010-2011	2014	15-40	27.9	837	16.7	-	-	-	-	^29^
Panidis D	Greece	NCEP-ATP III	-	2013	-	24.7	1223	15.8	-	31.3	277	10.1	^30^
Panidis D	Greece	AHA-NHLBI	-	2013	-	24.7	1223	23.9	-	31.3	277	18.8	^30^
Panidis D	Greece	IDF	-	2013	-	24.7	1223	28.9	-	31.3	277	23.8	^30^
Panidis D	Greece	Joint Defintion	-	2013	-	24.7	1223	29.5	-	31.3	277	23.8	^30^
Mandrelle K	India	Modified AHA ATP III	2009-2010	2012	19-38	26.1	120	37.5	-	-	-	-	^31^
Moini A	Iran	NCEP-ATP III	2008-2009	2012	15-40	28.0	282	22.7	-	-	-	-	^32^
Verit FF	Turkey	NCEP-ATP III	2004-2010	2012	18-34	26.0	163	25.7	-	26.3	53	26.3	^33^
Ishak A	Malaysia	IDF	2008-2010	2012	18-41	29.6	99	43.4	-	-	-	-	^34^
Bhattacharya SM	India	JS 2009	2007-2008	2011	-	17.0	96	60.8	-				^35^
Mehrabian F	Iran	NCEP-ATP III	2006-2008	2011	18-42	-	539	24.9	-	-	-	-	^36^
Hudecova M	Sweden	NCEP-ATP III	-	2011	15-46	43.0	84	23.8	-	43.7	87	8.0	^6^
VrbÍková J	Czech Republic	IDF	-	2011	22-28	16.8	43	11.6	22-27	17.5	48	2.1	^37^
Gangale MF	Italy	ATP III	-	2011	22-31		140	18.6	-	-	-	-	^38^
Hosseinpanah F	Iran	JS	2009-2010	2011	25-39	31.0	136	15.4	30-41	36.0	423	17.1	^39^
Dey R	India	NCEP-ATP III	2006–2007	2011	15-35		50	42.0	-	-	-	-	^40^
Bhattacharya SM	India	IDF	2004-2006	2010	15-40	22.1	198	47.5	-	-	-	-	^41^
Bhattacharya SM	India	ATP III	2004-2006	2010	-	22.2	198	37.9	-	-	-	-	^41^
Indhavivadhana S	Thailand	NCEP-ATP III	2007	2010	-	25.4	250	18.0	-	-	-	-	^42^
Indhavivadhana S	Thailand	IDF	2007	2010	-	25.4	250	21.2	-	-	-	-	^42^
Indhavivadhana S	Thailand	AHA/NHLBI	2007	2010	-	25.4	250	21.2	-	-	-	-	^42^
Fruzzetti F	Italy	Ferranti	2006-2007	2009	12-19	17.2	53	9.4	-	-	-	-	^43^
Moradi S	Iran	ATP III	-	2009	16-48	28.0	151	46.4	-	-	-	-	^44^
Ni R	China	IDF	2004-2008	2009	20-41	27.0	578	16.8	-	-	-	-	^45^
Gambineri A	Italy	NCEP-ATP III	-	2009	14-49	26.1	200	32.0	14-49	26.8	200	23.0	^46^
Gambineri A	Italy	IDF	-	2009	14-49	26.1	200	39.0	14-49	26.8	200	25.0	^46^
Gambineri A	Italy	AHA/NHLBI	-	2009	14-49	26.1	200	37.0	14-49	26.8	200	24.0	^46^
Soares EMM	Brazil	NCEP-ATP III	2004-2005	2008	20-34	26.4	102	28.4	-	-	-	-	^47^
Attaoua R	Romania	NCEP-ATP III	-	2008	19-57	23.1	107	15.8	-	34.1	100	4.0	^48^
Cheung LP	China	ATP III (Modified)	2003-7	2008	-	30.2	295	24.9	-	-	-	-	^49^
Cussons AJ	Australia	WHO	2000-5	2008	25-54	34.3	168	33.3	25-53	33.7	883	-	^50^
Cussons AJ	Australia	NCEP-ATP III	2000-5	2008	25-54	34.3	168	36.9	25-53	33.7	883	10.0	^50^
Cussons AJ	Australia	IDF	2000-5	2008	25-54	34.3	168	39.9	25-53	33.7	883	13.5	^50^
Gulcelik NE	Turkey	NCEP-ATP III	-	2008	-	24.6	30	33.3	-	26.1	60	11.7	^51^
Costa L	Brazil	NCEP-ATP III	2005-6	2007	19-38	24.1	90	30.4	19-38	30.9	44	6.8	^52^
Costa L	Brazil	IDF	2005-6	2007	19-38	24.1	90	32.6	19-38	30.9	44	9.1	^52^
Weerakiet S	Thailand	IDF	2002-5	2007	-	28.8	170	35.3	-	-	-	-	^53^
Marcondes JAM	Brazil	NCEP-ATP III	1995-2004	2007	-	25.0	73	38.4	-	-	-	-	^54^
Caliskan E	Turkey	NCEP-ATP III	2004-6	2007	-	23.2	182	8.2	-	23.6	182	2.7	^55^
Caliskan E	Turkey	IDF	2004-6	2007	-	23.2	182	14.3	-	23.6	182	2.7	^55^
Caliskan E	Turkey	WHO	2004-6	2007	-	23.2	182	8.2	-	23.6	182	2.7	^55^
Caliskan E	Turkey	AHA/NHLBI	2004-6	2007	-	23.2	182	10.4	-	23.6	182	6.6	^55^
Shroff R	USA	AHA	-	2007	-	32.0	24	25.0	-	24.0	36	17.0	^56^
Park HR	Korea	NCEP-ATP III	-	2007	16-39	26.0	113	14.5	30-80	46.9	774	4.3	^7^
Hahn S	Germany	NCEP-ATP III	-	2006	-	28.0	411	33.8	-	28.0	82	7.3	^57^
Carmina E	USA	ATP III	1991-2004	2006	18-40	24.9	282	8.2	-	25.2	85	2.4	^11^
Carmina E	USA	WHO	1991-2004	2006	18-40	24.9	282	16.0	-	25.2	85	2.4	^11^
Ehrmann DA	USA	ATP III	-	2006	18-41	28.4	368	33.4	-	-	-	-	^10^
Alvarez-Blasco F	Spain	ATP III	2002-5	2006	-	26.0	32	25.0	-	32.0	72	26.0	^58^
Coviello AD	USA	Cook	-	2006	14-19	17.0	49	37.0	-	-	-	-	^59^
Coviello AD	USA	Ferranti	-	2006	14-19	17.0	49	47.0	-	-	-	-	^59^
Leibel NL	USA	C-III	-	2006	12-19	16.0	36	19.4	-	-	-	-	^60^
Leibel NL	USA	C-04	-	2006	12-19	16.0	36	27.8	-	-	-	-	^60^
Apridonidze T	USA	NCEP-ATP III	2000-3	2005	20-40	29.9	106	43.0	-	-	-	-	^12^
Dokras A	USA	ATP III	2002	2005	18-49	28.0	129	47.3	18-50	44.0	177	6.8	^4^
Rabelo-Acevedo M	Puerto Rico	ATP III	-	2005	19-57	29.4	39	44.0	-	-	-	-	^61^
Vrbikova J	Czech Republic	ATP III	2001-3	2005	22-28	24.0	69	1.6	22-27	23.8	73	0	^37^
Vural B	Turkey	NCEP-ATP III	2002-4	2005	18-22	21.4	43	2.3	18-22	20.8	43	0	^62^
Vural B	Turkey	WHO	2002-4	2005	18-22	21.4	43	11.6	18-22	20.8	43	0	^62^
Faloia E	Italy	NCEP-ATP III	-	2004	-	22.0	50	8.0	-	-	-	-	^63^
Glueck CJ	USA	ATP III	-	2003	-	31.0	138	46.4	-	-	1887	22.8	^64^

**Table 3:** Prevalence of Metabolic Syndrome Components in Women with PCOS

**First Author**	**Country**	**Criteria**	**Study year**	**Publication Year**	**Characteristics of people with PCOS**								**Ref.**
					**Age range**	**Mean age**	**Total sample**	**Pr. WC (%)**	**Pr. HTN (%)**	**Pr. HDL (%)**	**Pr. FBS (%)**	**Pr. TG (%)**	
Kyrkou G	Greece	IDF	-	2015	14–44	24.7	230	72.2	12.6	26.1	7.0	10.4	^21^
Madani T	Iran	NCEP-ATP III	2012-2013	2015	-	28.6	624	34.6	2.2	71.5	13.1	26.0	^24^
Shabir I	India	IDF	2009-2010	2014	13-28	23.0	37	67.5	22.0	NE	36.0	48.0	^25^
Shabir I	India	ATP III	2009-2010	2014	13-28	23.0	37	67.5	22.0	NE	36.0	48.0	^25^
Rong Li	China	NCEP-ATP III	-	2014	19-45	29.1	833	84.8	45.7	85.9	55.0	63.4	^28^
Mandrelle K	India	Modified AHA ATP III	2009-2010	2012	19-38	26.1	120	45.8	20.0	91.7	8.3	-	^31^
Moini A	Iran	NCEP-ATP III	2008-2009	2012	15-40	28.0	282	31.0	10.6	68.8	3.2	33.0	^32^
Verit FF	Turkey	NCEP-ATP III	2004-2010	2012	18-34	26.0	163	26.4	17.8	42.3	12.3	22.1	^33^
Hudecova M	Sweden	NCEP-ATP III	-	2011	15-46	43.0	84	46.4	NE	NE	8.3	21.4	^6^
Hosseinpanah F	Iran	JIS	2009-2010	2011	18-45	31.0	136	81.0	NE	95.2	NE	87.7	^39^
Bhattacharya SM	India	IDF	2004-2006	2010	-	22.1	198	NE	68.1	98.9	68.1	98.9	^35^
Bhattacharya SM	India	ATP III	2004-2006	2010	-	22.2	198	NE	52.0	98.7	52.0	98.7	^35^
Indhavivadhana S	Thailand	NCEP-ATP III	2007	2010	-	25.4	250	48.8	14.0	39.6	6.8	17.2	^42^
Indhavivadhana S	Thailand	IDF	2007	2010	-	25.4	250	48.8	14.0	39.6	6.8	17.2	^42^
Indhavivadhana S	Thailand	AHA/NHLBI	2007	2010	-	25.4	250	48.8	14.0	39.6	6.8	17.2	^42^
Fruzzetti F	Italy	Ferranti	2006-2007	2009	12-19	17.2	53	28.3	28.3	43.4	1.9	7.5	^43^
Moradi S	Iran	ATP III	-	2009	16-48	28.0	151	55.6	23.0	71.0	7.3	48.0	^44^
Ni R	China	IDF	2004-2008	2009	20-41	27.0	578	38.4	16.1	41.6	19.8	41.6	^45^
Gambineri A	Italy	NCEP-ATP III	-	2009	14-49	26.1	200	57.0	50.0	58.0	6.0	11.0	^46^
Gambineri A	Italy	IDF	-	2009	14-49	26.1	200	57.0	50.0	58.0	17.0	11.0	^46^
Gambineri A	Italy	AHA/NHLBI	-	2009	14-49	26.1	200	57.0	50.0	58.0	17.0	11.0	^46^
Soares EMM	Brazil	NCEP-ATP III	2004-2005	2008	20-34	26.4	102	57.9	18.6	69.6	2.9	31.7	^47^
Cheung LP	China	ATP III (Modified)	2003-7	2008	-	30.2	295	53.1	29.4	28.6	21.4	21.4	^49^
Gulcelik NE	Turkey	NCEP-ATP III	-	2008	-	24.6	30	21.0	6.0	48.0	2.0	17.0	^51^
Costa L	Brazil	NCEP-ATP III	2005-6	2007	19-38	24.1	90	47.8	28.2	52.2	4.3	8.7	^52^
Costa L	Brazil	IDF	2005-6	2007	19-38	24.1	90	47.8	28.2	52.2	4.3	8.7	^52^
Weerakiet S	Thailand	IDF	2002-5	2007	-	28.8	170	55.9	28.2	59.4	23.5	25.9	^53^
Marcondes JAM	Brazil	NCEP-ATP III	1995-2004	2007	-	25.0	73	49.3	24.7	67.6	6.9	31.8	^54^
Caliskan E	Turkey	NCEP-ATP III	2004-6	2007	-	23.2	182	19.2	15.4	61.0	5.5	5.5	^55^
Caliskan E	Turkey	IDF	2004-6	2007	-	23.2	182	19.2	15.4	61.0	5.5	5.5	^55^
Caliskan E	Turkey	WHO	2004-6	2007	-	23.2	182	19.2	15.4	61.0	5.5	5.5	^55^
Caliskan E	Turkey	AHA/NHLBI	2004-6	2007	-	23.2	182	19.2	15.4	61.0	5.5	5.5	^55^
Park HR	Korea	NCEP-ATP III	-	2007	16-39	26.0	113	24.0	20.2	45.1	0.9	13.3	^7^
Hahn S	Germany	NCEP-ATP III	-	2006	-	28.0	411	74.4	45.5	44.8	15.1	23.4	^57^
Carmina E	USA	ATP III	1991-2004	2006	18-40	24.9	282	39.0	7.3	45.1	3.1	9.3	^11^
Carmina E	USA	WHO	1991-2004	2006	18-40	24.9	282	39.0	7.3	45.1	3.1	9.3	^11^
Ehrmann DA	USA	ATP III	-	2006	18-41	28.4	368	80.0	21.0	66.0	5.0	32.0	^10^
Alvarez-Blasco F	Spain	ATP III	2002-5	2006	-	26.0	32	66.0	25.0	72.0	6.0	19.0	^58^
Coviello AD	USA	Cook	-	2006	14-19	17.0	49	47.0	41.0	84.0	2.0	49.0	^59^
Coviello AD	USA	Ferranti	-	2006	14-19	17.0	49	65.0	41.0	84.0	2.0	53.0	^59^
Apridonidze T	USA	NCEP-ATP III	2000-3	2005	20-39	29.9	106	67.0	45.0	68.0	3.8	35.0	^12^
Rabelo-Acevedo M	Puerto Rico	ATP III	-	2005	19-57	29.4	39	89.5	36.0	71.0	-	43.0	^61^
Vrbikova J	Czech Republic	ATP III	2001-3	2005	22-28	24.0	64	11.0	13.0	34.8	0.0	5.8	^37^
Glueck CJ	USA	ATP III	-	2003	-	31.0	138	85.5	44.9	64.5	5.1	32.6	^64^

### Prevalence of MetS and its component in women with PCOS

**Figure 2:**
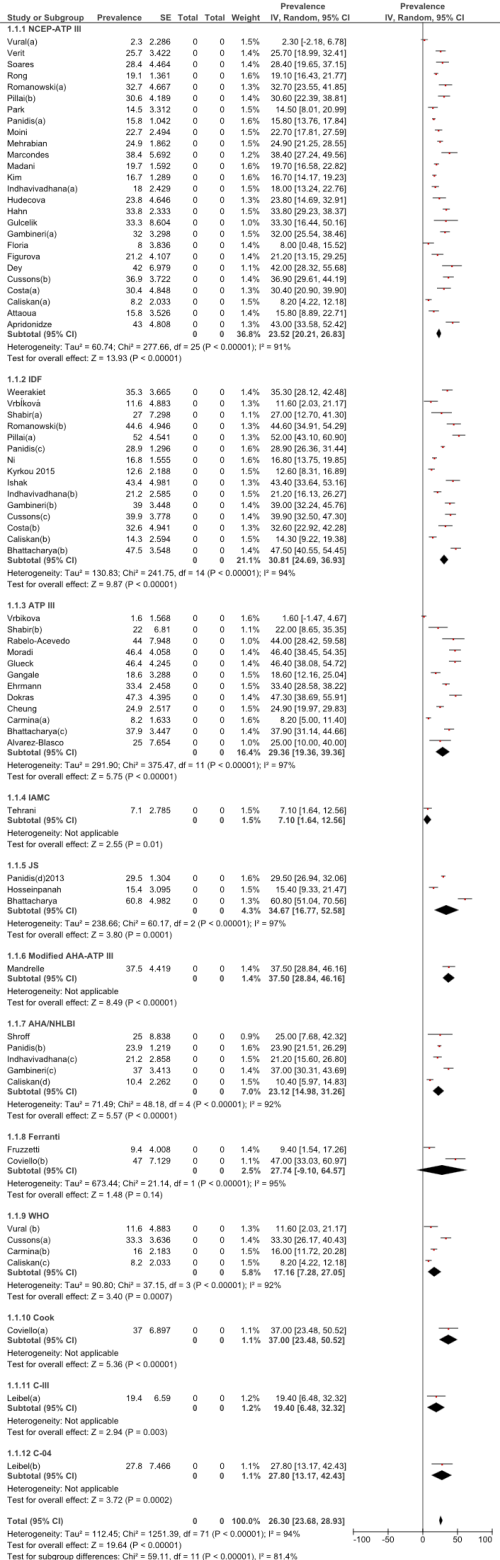
Forest plot of MetS prevalence among women with PCOS by definition/criteria.

The pooled prevalence of MetS among PCOS women was found to be 26.30% (95% CI: 23.68–28.93). However, the pooled prevalence differed according to the definition of MetS used and were as follows: NCEP-ATP III (23.52%, 95% CI:20.21-26.83); IDF (30.81, 95% CI: 24.69–36.93); ATP III (29.36, 95% CI: 19.36–39.36); IMAC (7.10 , 95% CI: 1.64–12.56); JS (34.67, 95% CI: 16.77–52.58); Modified AHA-ATPIII (37.50, 95% CI: 28.84–46.16); AHA-NHLBI (23.12, 95% CI: 14.98–31.26); Ferranti (27.74%, 95% CI: 9.10–64.57); WHO (17.16, 95% CI: 7.28–27.05); Cook (37.00, 95% CI: 23.48–50.52); C-III (19.40, 95% CI: 6.48–32.32); and C-04 (27.80, 95% CI: 13.17–42.43) (Figure 2).

The pooled prevalence of MetS components in women diagnosed with PCOS are presented in online Figure S1-S5 (See Supplementary file 1). The prevalence of the individual components of MetS in women diagnosed with PCOS were: high FBS - 13.44% (95% CI: 9.05–17.84), low HDL - 61.87% (95% CI: 53.31–70.43), HTN - 26.69% (95% CI: 20.34–33.3), high TG - 33.09% (95% CI: 18.82–47.35) and high WC - 52.23% (95% CI: 43.84–60.61).

### Association between PCOS and MetS

**Figure 3:**
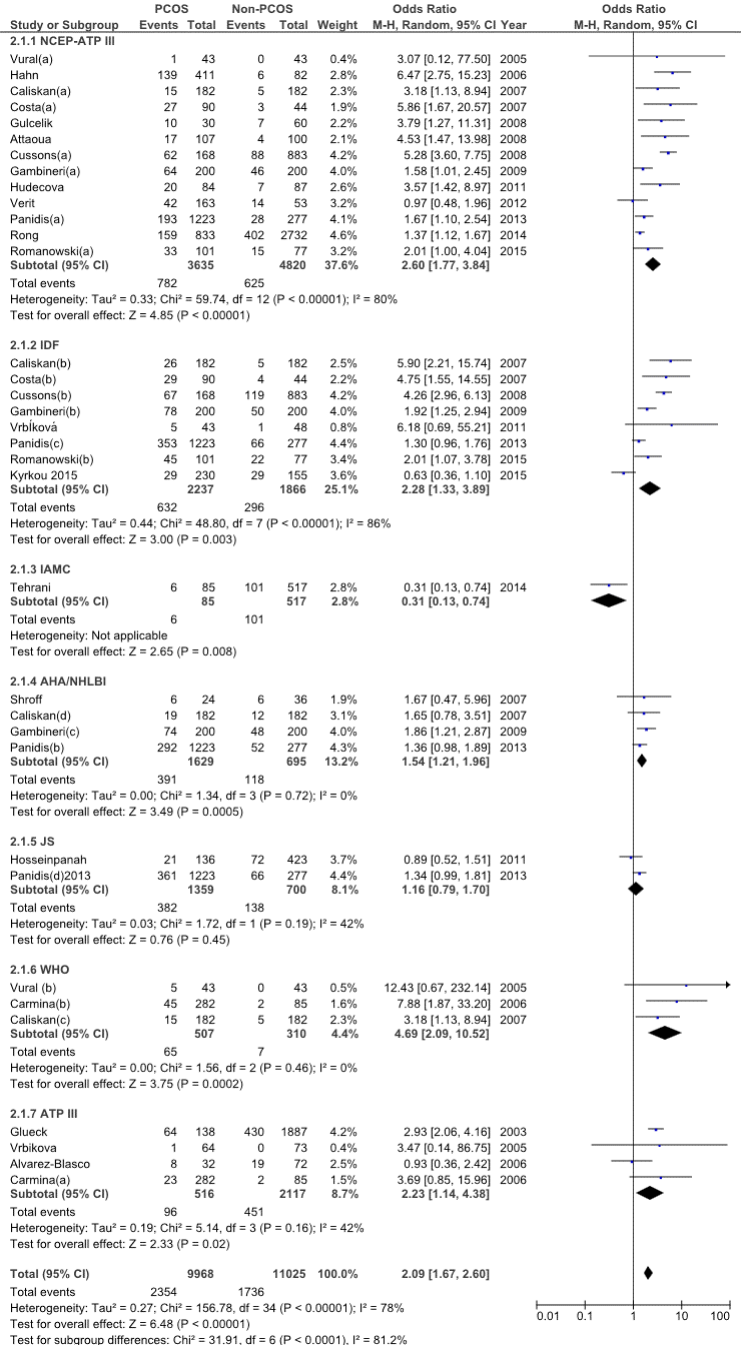
Forest plot of MetS risk among patients with PCOS compared with healthy controls, by definition/criteria.

This meta-analysis also estimated the odds of MetS in woman diagnosed with PCOS and compared the prevalence of MetS with healthy women (i.e., not diagnosed with PCOS) using comparative cross-sectional studies. Additionally, the association between PCOS and MetS was examined using OR. In general, the odds of being diagnosed with MetS increased two fold for those diagnosed with PCOS (OR=2.09, 95% CI: 1.67–2.60), in comparison with the healthy controls. This OR varied widely, according to the MetS definition used, and included the following: NCEP-ATP III - 2.60 (95% CI: 1.77–3.84); IDF - 2.28 (95% CI: 1.33–3.89); IAMC - 0.31(95% CI: 0.13–0.74); AHA-NHLBI - 1.54 (95% CI: 1.21–1.96); JS - 1.16 (95% CI: 0.79–1.70); WHO - 4.69 (95% CI: 2.09–10.52); and ATP III - 2.23 (95% CI: 1.14–4.38) (Figure 3).

### Heterogeneity and Meta-regression

Due to the significant heterogeneity in the ORs reported by the different studies (χ^2^=1251.39 *df*=71 *P*<0.00001 and I^2^=94%), a number of variables were entered into a meta-regression model in order to identify the source (s) of the heterogeneity. *Criteria*, *publication date*, *continent* and *age* were examined in this model, but only *criteria* and *Publication date* were statistically significant (*P*<0.001) (Table 4).

**Table 4:** Association between studied covariates with the MetS odds ratio among PCOS patients, compared to healthy controls

**Variables**	**Meta-regression**	
	**Univariate**	**Multivariate** ^a^
	***P*** ** value**	***P*** ** value**
Criteria	0.004	0.001
Publication date	<0.001	<0.001
Continent	0.95	
Mean age	0.68	-

^a^Between-study variance assessed by moment-based estimate (tau2= 0.22).

Therefore, a subgroup analysis was conducted using publication date and diagnostic criteria used. The subgroup analysis also confirmed the results of the meta-regression, in that the OR for the relationship between PCOS and MetS was found to be different according to the research period, with studies conducted during 2003-2010 having a higher OR (OR = 3.02; 95% CI: 2.32-3.93) than those conducted from 2011-2015 (OR = 1.27; 95% CI: 1.03-1.58). Interestingly, the more recent studies reported a weaker association between PCOS and MetS than the earlier studies.

### Publication bias

**Figure 4:**
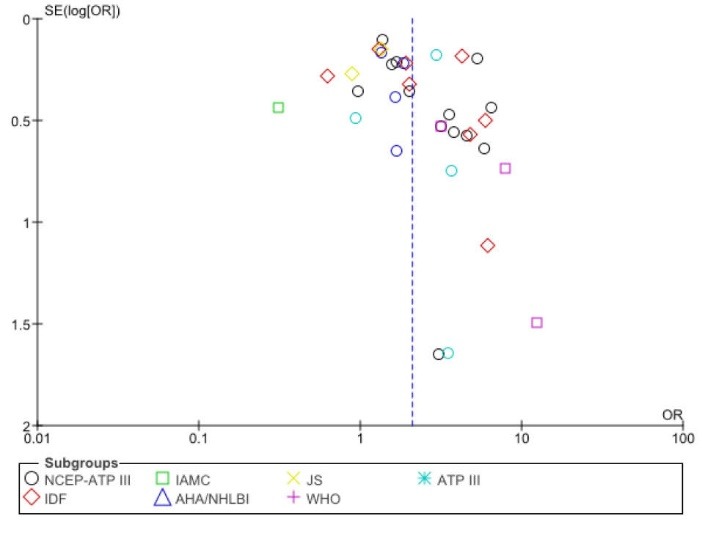
Funnel plot assessing the publication bias on the association between MetS and PCOS.

The publication bias in relation to the OR for MetS among women diagnosed with PCOS (compared to the healthy controls) was examined using funnel plots and Egger’s test. Figure 4 shows that there was no significant publication bias (*P*=0.112). Notably, the risk of bias assessment showed that the majority of the studies included had acceptable validity and no study was found to have a score lower than 3 (Table S1, online Supplementary file 1).

## Discussion

This study found that the general prevalence of MetS in women with PCOS varied according to the definition used. The highest prevalence of 37.50% was identified using the modified AHA ATP III definition, while the lowest prevalence was 7.10%, using the IAMC definition. Similar differences have also been observed in other studies.^30,55^

Using a cross-sectional study, the prevalence of MetS in patients with PCOS, based on the WHO criteria, was found to be 33%. However, using the NCEP-ATP-III and IDF criteria, these estimates were found to be 37% and 40%, respectively.^50^ These findings support previous research, which also found the IDF criteria to be more sensitive than the NCEP-ATP III criteria in identifying PCOS subjects with MetS (52% vs. 30.6%).^23^ The present findings also supported research by Bhattacharya, which found the prevalence of MetS among women with PCOS to be 37.9% and 47.5% using the NCEP-ATP III and IDF criteria, respectively.^65^ In another study, which used four definitions of MetS (NCEP-WHO-AHA/NCLBI-IDF), the IDF definition was again found to be the most sensitive in identifying MetS cases. This might be the result of the lower values of both waist circumference and FBS levels used in the IDF criteria.^55^

The present study found a significant relationship between PCOS and MetS (OR=2.09), which ranged from 0.31 to 4.69, depending upon the definition of MetS used. This association has been examined previously by a number of studies.^30,50,55^ For instance, research using the NCEP-ATP III definition reported a higher prevalence of MetS in women suffering from PCOS than among healthy controls.^30^ The same study also reported that there was no significant relationship between PCOS and MetS when using the AHA/NHLBI, IDF and Joint definitions.^30^ Moreover, Çalışkan et al. showed a greater prevalence of MetS among patient with PCOS, than in the control group, when using all criteria except for AHA/NCLBI.^55^ In addition, another study reported a 4-fold increased prevalence of MetS in PCOS-suffering women, compared to the overall population.^50^ Previous research has also found a much greater likelihood (4.2- fold) of developing MetS among adolescent Indian girls with PCOS, when compared to those without PCOS.^35^ In contrast, Hosseinpanah et al did not find a significantly higher frequency of MetS in a sample of Iranian women with PCOS, than that found in healthy subjects.^39^

Therefore, there is great variation in the prevalence of MetS, even among studies which used the same definition of PCOS. This variability is likely to be due to the following reasons: i) the cut off points used in the different definitions; and ii) inconsistency in the number of elements required by each definition. Consequently, a general and diagnostic definition is required for planning early prevention and for the identification of MetS-susceptible PCOS patients. It is also unclear which definition(s) is/are the best. Some of the previously conducted studies have reported the ATP III to be better than the IDF criteria, in terms of prediction.^66,67^ In contrast, Tong et al. highlighted the inability of the IDF definition for identifying MetS patients with a high risk of coronary heart disease.^68^

Previous research has reported that the relationship between PCOS and MetS to be independent of age. In support of previous research, we also found that the heterogeneity in the relationship between PCOS and MetS could not be explained by the participants’ age. Similarly, Vural et al indicated a higher frequency of MetS among women with PCOS in all age groups.^62^ In contrast, a cross-sectional study reported MetS prevalence of 12.1% for women aged 20-24 years old, 31.7% among 25-29 year olds and 42.9% among those aged 30-34 years old.^47^ Also, several other studies have found the prevalence of MetS to be heavily age-dependent.^69,70^ A higher risk of MetS has also been reported among women under 30 years old with PCOS, which highlights the importance of early and regular screening for MetS among young women with PCOS.^65^

In our study the prevalence of MetS components (e.g. WC, and HTN) were estimated among women with PCOS. This found a high prevalence of WC among these women (52.23%). In previous research, the prevalence of obesity in women with PCOS has been reported to be 30%–75%,^71,72^ which is extremely high and demonstrates the strong effect of adiposity on the development and maintenance of PCOS.^73^

The current study also found that the various indicators of MetS, such as high levels of TG and FBS and a low HDL level, were more prevalent among women with PCOS than among the healthy controls. This finding supports previous research which has also found higher rates of MetS components among women with PCOS, than among healthy controls.^35,74,75^ For instance, research in India found a dyslipidemia rate of 90.2% among adolescent women with PCOS and 21.6% of their sample had high levels of FBS.^35^ Furthermore, a meta-analysis found higher levels of low-density lipoprotein in women with PCOS, than among healthy controls.^74^ Several studies have also reported dyslipidemia to be the most frequently identified indicator of metabolic disorder among patients with PCOS, with prevalence rates of up to 70% being reported.^5,12,75^ The high prevalence of this symptom is thought to be as a result of changes in the concentration of several hormones (insulin, estrogens, and androgens) among women with PCOS, which alters the metabolism of lipoproteins.^76^ In an effort to remove excess hyperandrogenism and estrogen in women with PCOS, using gonadotrophin‐releasing hormone agonists (GnRHa), research found that after three months of treatment androgen and estrogen levels were reduced and a slight reduction was also found in the levels of triglyceride.^77^ In contrast, Pirwany et al indicated that metabolic disorder was more closely related to adiposity/insulin metabolism than to circulating androgen levels.^78^ In general, because of a higher prevalence of dyslipidemia among the PCOS patients, it is important that the levels of serum lipids should be carefully monitored.

This study is a comprehensive systematic review and meta-analysis on the prevalence of MetS, and its components, among women diagnosed with PCOS. An extensive search of 10 databases was made in order to avoid missing any relevant information. However, as with any study this meta-analysis and systematic review had a number of strengths and limitations. The first strength of this study was the comprehensive search strategy which covered 10 databases. In addition, the search and data extraction processes were conducted independently by two authors, reducing the chances that something would be missed. Furthermore, the prevalence of MetS was presented by calculating the ORs using different diagnostic criteria, rather than relying solely on one. Finally, another strength of the study was that the possible sources of heterogeneity across studies were examined using a series of meta-regression analyses.

This study also had a number of limitations, including the fact that non-English studies were not included and that surprisingly there were no studies identified from Africa. Finally, due to sparse data bias, subgroup analysis on the different variables could not be undertaken.

## Conclusion

The present study found that women with PCOS had a much higher prevalence of MetS than was found among healthy controls. Therefore, the present study highlights the importance of preventive strategies designed to prevent MetS among women with PCOS. Furthermore, as low HDL and high WC were the most commonly identified components of MetS, among women diagnosed with PCOS, these two components particularly need to be carefully addressed in prevention strategies.

## Ethical approval

The present study is based on published data, and hence ethical approval was not required.

## Competing interests

The authors declare that they have no competing interests.

## Acknowledgements

This study was funded by the Vice Chancellor for Research and Technology of Maragheh University of Medical Sciences.

## Supplementary Materials

Supplementary file 1 contains Figures S1-S5 and Table S1.
