# Supplementary material for: Metabolic syndrome and its components among women with polycystic ovary syndrome: a systematic review and meta-analysis
Source: J Cardiovasc Thorac Res. 2018 May 28;10(2):56–69. doi: 10.15171/jcvtr.2018.10 (PMC6088762; doi:10.15171/jcvtr.2018.10)
Supplement: Supplementary file 1 — contains Figures S1-S5 and Table S1. [file jcvtr-10-56-s001.pdf]

Study or Subgroup	WC	SE	Total	Total	Weight	WC IV, Random, 95% CI
Alvarez-Blasco	66	8.374	0	0	2.9%	66.00 [49.59, 82.41]
Apridonidze	67	4.567	0	0	3.1%	67.00 [58.05, 75.95]
Caliskan(a)	19.2	2.919	0	0	3.2%	19.20 [13.48, 24.92]
Carmina(a)	39	2.904	0	0	3.2%	39.00 [33.31, 44.69]
Cheung	53.1	2.905	0	0	3.2%	53.10 [47.41, 58.79]
Costa(a)	47.8	5.265	0	0	3.1%	47.80 [37.48, 58.12]
Coviello(a)	47	7.129	0	0	3.0%	47.00 [33.03, 60.97]
Coviello(b)	65	6.813	0	0	3.0%	65.00 [51.65, 78.35]
Ehrmann	80	2.085	0	0	3.2%	80.00 [75.91, 84.09]
Fruzzetti	28.3	6.187	0	0	3.0%	28.30 [16.17, 40.43]
Gambineri(a)	57	3.5	0	0	3.2%	57.00 [50.14, 63.86]
Glueck	85.5	2.99	0	0	3.2%	85.50 [79.64, 91.36]
Gulcelik	21	7.436	0	0	3.0%	21.00 [6.43, 35.57]
Hahn	74.4	2.152	0	0	3.2%	74.40 [70.18, 78.62]
Hosseinpanah	81	3.363	0	0	3.2%	81.00 [74.41, 87.59]
Hudecova	46.4	5.441	0	0	3.1%	46.40 [35.74, 57.06]
Indhavivadhana(a)	48.8	3.161	0	0	3.2%	48.80 [42.60, 55.00]
Kyrkou 2015	72.2	2.954	0	0	3.2%	72.20 [66.41, 77.99]
Madani	34.6	1.904	0	0	3.2%	34.60 [30.87, 38.33]
Mandrelle	45.8	4.548	0	0	3.1%	45.80 [36.89, 54.71]
Marcondes	49.3	5.851	0	0	3.1%	49.30 [37.83, 60.77]
Moini	31	2.754	0	0	3.2%	31.00 [25.60, 36.40]
Moradi	55.6	4.043	0	0	3.1%	55.60 [47.68, 63.52]
Ni	38.4	2.022	0	0	3.2%	38.40 [34.44, 42.36]
Park	24	4.017	0	0	3.1%	24.00 [16.13, 31.87]
Rabelo-Acevedo	89.5	4.908	0	0	3.1%	89.50 [79.88, 99.12]
Rong	84.8	1.243	0	0	3.2%	84.80 [82.36, 87.24]
Shabir(a)	67.5	7.7	0	0	2.9%	67.50 [52.41, 82.59]
Soares	57.9	4.888	0	0	3.1%	57.90 [48.32, 67.48]
Verit	26.4	3.452	0	0	3.2%	26.40 [19.63, 33.17]
Vrbikova	11	3.911	0	0	3.2%	11.00 [3.33, 18.67]
Weerakiet	55.9	3.808	0	0	3.2%	55.90 [48.44, 63.36]
**Total (95% CI)**			**0**	**0**	**100.0%**	**52.23 [43.84, 60.61]**

Heterogeneity: $Tau^2 = 564.76$; $Chi^2 = 1777.70$, df = 31 ($P < 0.00001$); $I^2 = 98\%$
Test for overall effect: $Z = 12.21$ ($P < 0.00001$)

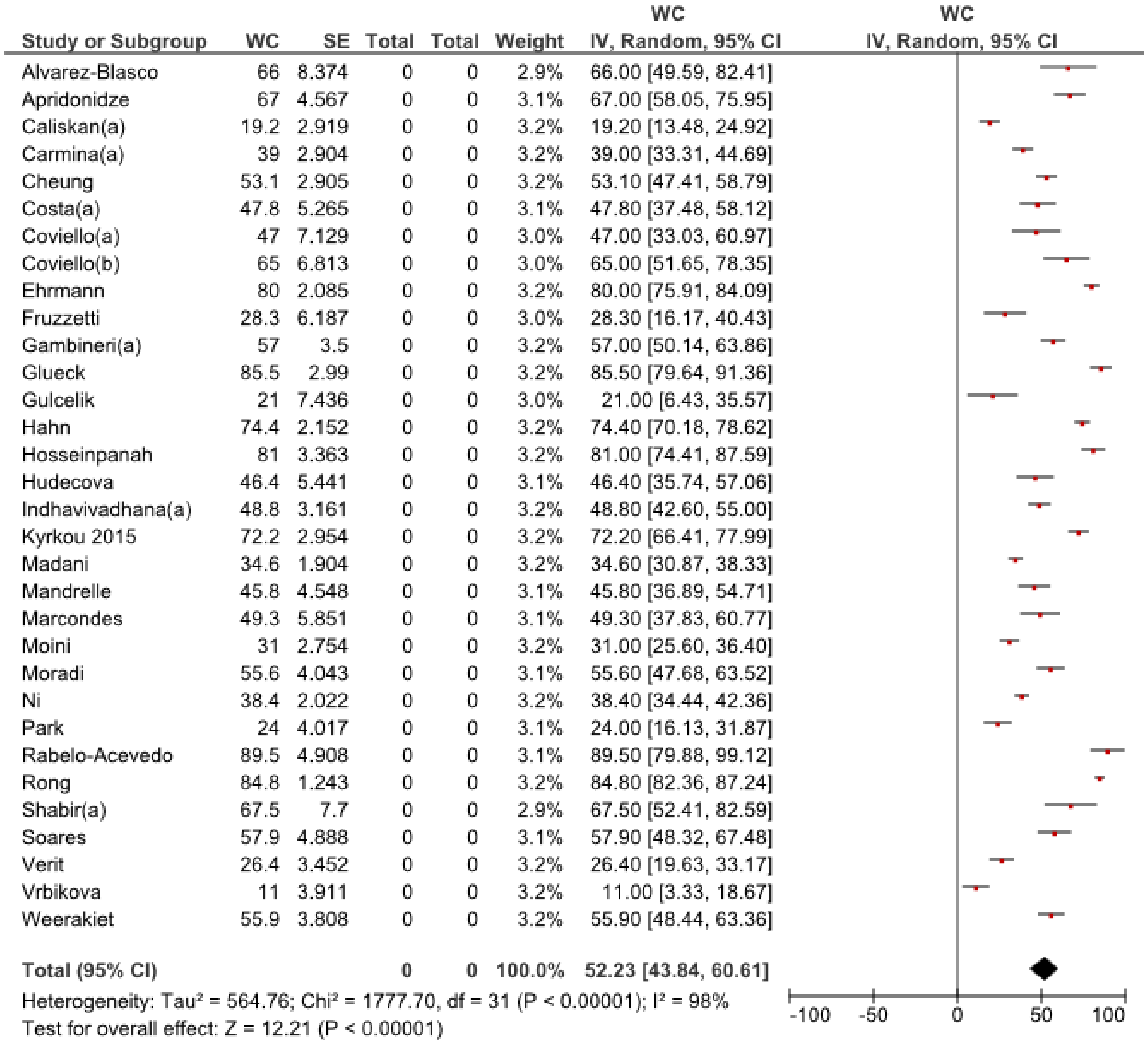

Figure S1. Forest plot of the prevalence of High WC among women with polycystic ovary syndrome

Study or Subgroup	Prevalence	SE	Total	Total	Weight	Prevalence IV, Random, 95% CI
Alvarez-Blasco	25	7.654	0	0	2.9%	25.00 [10.00, 40.00]
Apridonidze	45	4.832	0	0	3.2%	45.00 [35.53, 54.47]
Bhattacharya(b)	68.1	3.312	0	0	3.3%	68.10 [61.61, 74.59]
Bhattacharya(c)	52	3.55	0	0	3.3%	52.00 [45.04, 58.96]
Caliskan(a)	15.4	2.675	0	0	3.3%	15.40 [10.16, 20.64]
Carmina(a)	7.3	1.549	0	0	3.4%	7.30 [4.26, 10.34]
Cheung	29.4	2.652	0	0	3.3%	29.40 [24.20, 34.60]
Costa(a)	28.2	4.743	0	0	3.2%	28.20 [18.90, 37.50]
Coviello(a)	41	7.026	0	0	2.9%	41.00 [27.23, 54.77]
Ehrmann	21	2.123	0	0	3.3%	21.00 [16.84, 25.16]
Fruzzetti	28.3	6.187	0	0	3.0%	28.30 [16.17, 40.43]
Gambineri(a)	50	3.535	0	0	3.3%	50.00 [43.07, 56.93]
Glueck	44.9	4.234	0	0	3.2%	44.90 [36.60, 53.20]
Gulcelik	6	4.335	0	0	3.2%	6.00 [-2.50, 14.50]
Hahn	45.5	2.456	0	0	3.3%	45.50 [40.69, 50.31]
Indhavivadhana(a)	14	2.194	0	0	3.3%	14.00 [9.70, 18.30]
Kyrkou 2015	12.6	2.188	0	0	3.3%	12.60 [8.31, 16.89]
Madani	2.2	0.587	0	0	3.4%	2.20 [1.05, 3.35]
Mandrelle	20	3.651	0	0	3.3%	20.00 [12.84, 27.16]
Marcondes	24.7	5.047	0	0	3.1%	24.70 [14.81, 34.59]
Moini	10.6	1.833	0	0	3.4%	10.60 [7.01, 14.19]
Moradi	23	3.424	0	0	3.3%	23.00 [16.29, 29.71]
Ni	16.1	1.528	0	0	3.4%	16.10 [13.11, 19.09]
Park	20.2	3.776	0	0	3.2%	20.20 [12.80, 27.60]
Rabelo-Acevedo	36	7.686	0	0	2.9%	36.00 [20.94, 51.06]
Rong	45.7	1.725	0	0	3.4%	45.70 [42.32, 49.08]
Shabir(a)	22	6.81	0	0	3.0%	22.00 [8.65, 35.35]
Soares	18.6	3.852	0	0	3.2%	18.60 [11.05, 26.15]
Verit	17.8	2.996	0	0	3.3%	17.80 [11.93, 23.67]
Vrbikova	13	4.203	0	0	3.2%	13.00 [4.76, 21.24]
Weerakiet	28.2	3.451	0	0	3.3%	28.20 [21.44, 34.96]
**Total (95% CI)**			**0**	**0**	**100.0%**	**26.69 [20.34, 33.03]**

Heterogeneity: Tau² = 308.60; Chi² = 1635.45, df = 30 (P < 0.00001); I² = 98%
Test for overall effect: Z = 8.24 (P < 0.00001)

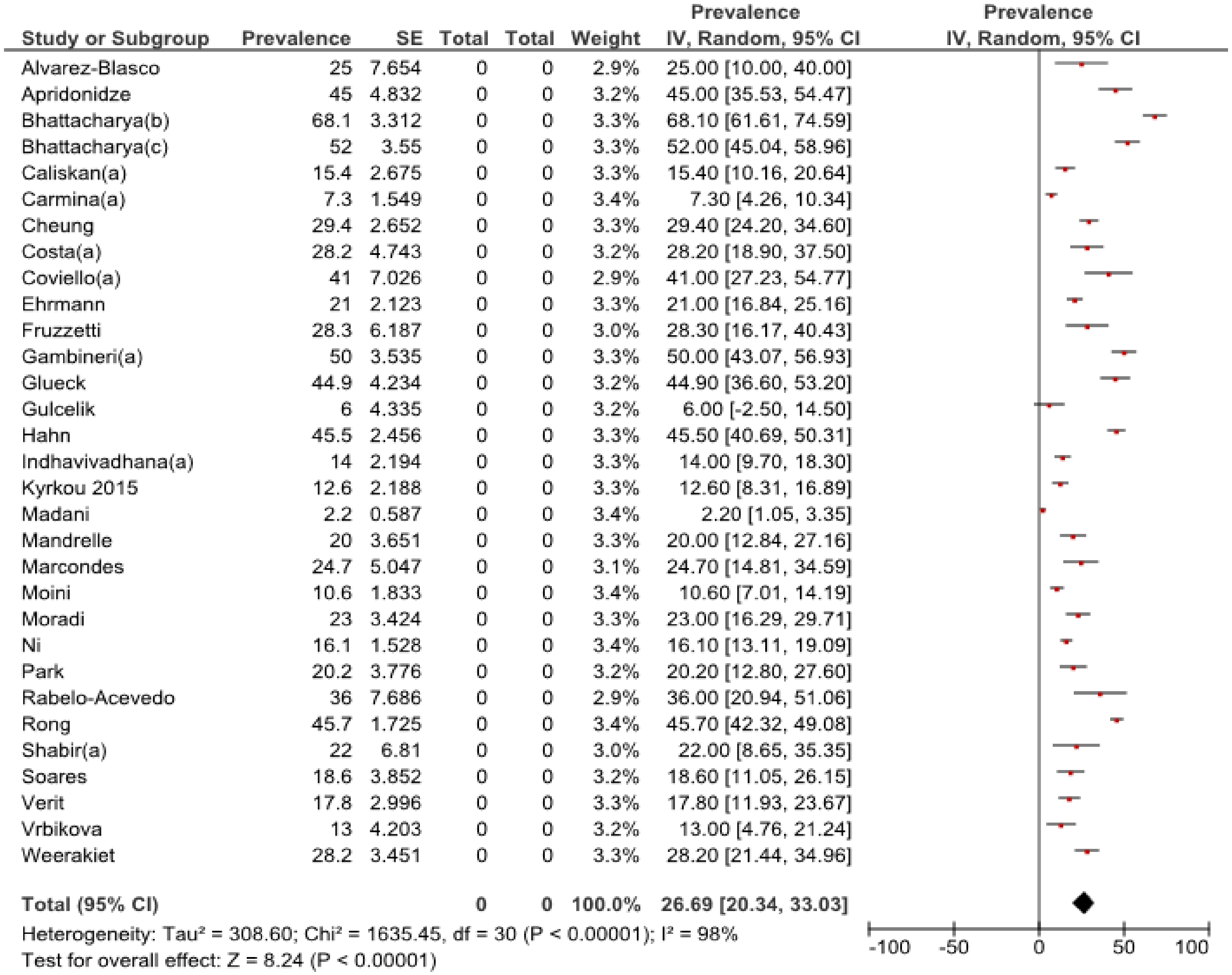

Figure S2. Forest plot of the prevalence of HTN among women with polycystic ovary syndrome.

Study or Subgroup	Prevalence	SE	Total	Total	Weight	Prevalence IV, Random, 95% CI	Prevalence IV, Random, 95% CI
Alvarez-Blasco	6	4.198	0	0	2.9%	6.00 [-2.23, 14.23]	
Apridonidze	3.8	1.857	0	0	3.2%	3.80 [0.16, 7.44]	
Bhattacharya	68.1	3.312	0	0	3.0%	68.10 [61.61, 74.59]	
Bhattacharya(b)	52	3.55	0	0	3.0%	52.00 [45.04, 58.96]	
Caliskan(a)	5.5	1.689	0	0	3.2%	5.50 [2.19, 8.81]	
Carmina(a)	3.1	1.032	0	0	3.2%	3.10 [1.08, 5.12]	
Cheung	21.4	2.387	0	0	3.1%	21.40 [16.72, 26.08]	
Costa(a)	4.3	2.138	0	0	3.2%	4.30 [0.11, 8.49]	
Coviello(a)	2	2	0	0	3.2%	2.00 [-1.92, 5.92]	
Ehrmann	5	1.136	0	0	3.2%	5.00 [2.77, 7.23]	
Fruzzetti	1.9	1.875	0	0	3.2%	1.90 [-1.77, 5.57]	
Gambineri(a)	6	1.679	0	0	3.2%	6.00 [2.71, 9.29]	
Gambineri(b)	17	2.656	0	0	3.1%	17.00 [11.79, 22.21]	
Gambineri(c)	11	2.212	0	0	3.2%	11.00 [6.66, 15.34]	
Glueck	5.1	1.872	0	0	3.2%	5.10 [1.43, 8.77]	
Gulcelik	2	2.556	0	0	3.1%	2.00 [-3.01, 7.01]	
Hahn	15.1	1.766	0	0	3.2%	15.10 [11.64, 18.56]	
Hudecova	8.3	3.01	0	0	3.1%	8.30 [2.40, 14.20]	
Indhavivadhana(a)	6.8	1.592	0	0	3.2%	6.80 [3.68, 9.92]	
Kyrkou 2015	7	1.682	0	0	3.2%	7.00 [3.70, 10.30]	
Madani	13.1	1.35	0	0	3.2%	13.10 [10.45, 15.75]	
Mandrelle	8.3	2.518	0	0	3.1%	8.30 [3.36, 13.24]	
Marcondes	6.9	2.966	0	0	3.1%	6.90 [1.09, 12.71]	
Moini	3.2	1.048	0	0	3.2%	3.20 [1.15, 5.25]	
Moradi	7.3	2.116	0	0	3.2%	7.30 [3.15, 11.45]	
Ni	19.8	1.657	0	0	3.2%	19.80 [16.55, 23.05]	
Park	0.9	0.888	0	0	3.2%	0.90 [-0.84, 2.64]	
Rong	55	1.723	0	0	3.2%	55.00 [51.62, 58.38]	
Shabir(a)	36	7.891	0	0	2.3%	36.00 [20.53, 51.47]	
Soares	2.9	1.661	0	0	3.2%	2.90 [-0.36, 6.16]	
Verit	12.3	2.572	0	0	3.1%	12.30 [7.26, 17.34]	
Weerakiet	23.5	3.251	0	0	3.0%	23.50 [17.13, 29.87]	
**Total (95% CI)**			**0**	**0**	**100.0%**	**13.44 [9.05, 17.84]**	

Heterogeneity: Tau² = 154.56; Chi² = 1547.60, df = 31 (P < 0.00001); I² = 98%

Test for overall effect: Z = 5.99 (P < 0.00001)

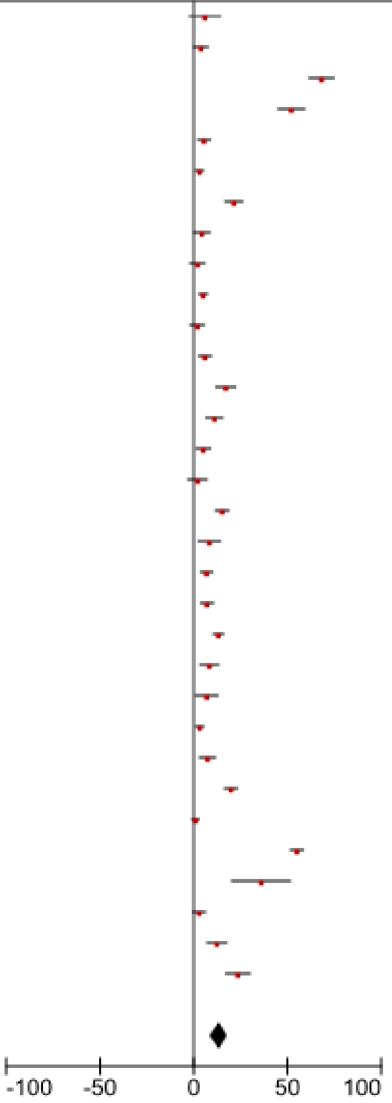

Figure S3. Forest plot of the prevalence of High FBS among women with polycystic ovary syndrome.

Study or Subgroup	Prevalence	SE	Total	Total	Weight	Prevalence IV, Random, 95% CI
Alvarez-Blasco	19	6.934	0	0	3.0%	19.00 [5.41, 32.59]
Apridonidze	35	4.632	0	0	3.0%	35.00 [25.92, 44.08]
Bhattacharya	98.9	0.741	0	0	3.1%	98.90 [97.45, 100.35]
Bhattacharya(b)	98.7	0.805	0	0	3.1%	98.70 [97.12, 100.28]
Caliskan(a)	5.5	1.689	0	0	3.1%	5.50 [2.19, 8.81]
Carmina(a)	9.3	1.729	0	0	3.1%	9.30 [5.91, 12.69]
Cheung	21.4	2.387	0	0	3.1%	21.40 [16.72, 26.08]
Costa(a)	8.7	2.97	0	0	3.0%	8.70 [2.88, 14.52]
Coviello(a)	49	7.141	0	0	3.0%	49.00 [35.00, 63.00]
Coviello(b)	53	7.129	0	0	3.0%	53.00 [39.03, 66.97]
Ehrmann	32	2.431	0	0	3.1%	32.00 [27.24, 36.76]
Fruzzetti	7.5	3.617	0	0	3.0%	7.50 [0.41, 14.59]
Gambineri(a)	11	2.212	0	0	3.1%	11.00 [6.66, 15.34]
Glueck	32.6	3.99	0	0	3.0%	32.60 [24.78, 40.42]
Gulcelik	17	6.858	0	0	3.0%	17.00 [3.56, 30.44]
Hahn	23.4	2.088	0	0	3.1%	23.40 [19.31, 27.49]
Hosseinpanah	87.7	2.816	0	0	3.0%	87.70 [82.18, 93.22]
Hudecova	21.4	4.474	0	0	3.0%	21.40 [12.63, 30.17]
Indhavivadhana(a)	17.2	2.386	0	0	3.1%	17.20 [12.52, 21.88]
Kyrkou 2015	10.4	2.012	0	0	3.1%	10.40 [6.46, 14.34]
Madani	26	1.755	0	0	3.1%	26.00 [22.56, 29.44]
Marcondes	31.8	5.45	0	0	3.0%	31.80 [21.12, 42.48]
Moini	33	2.8	0	0	3.0%	33.00 [27.51, 38.49]
Moradi	48	4.065	0	0	3.0%	48.00 [40.03, 55.97]
Ni	41.6	2.05	0	0	3.1%	41.60 [37.58, 45.62]
Park	13.3	3.194	0	0	3.0%	13.30 [7.04, 19.56]
Rabelo-Acevedo	43	7.927	0	0	3.0%	43.00 [27.46, 58.54]
Rong	63.4	1.669	0	0	3.1%	63.40 [60.13, 66.67]
Shabir(a)	48	8.213	0	0	2.9%	48.00 [31.90, 64.10]
Soares	31.7	4.607	0	0	3.0%	31.70 [22.67, 40.73]
Verit	22.1	3.249	0	0	3.0%	22.10 [15.73, 28.47]
Vrbikova	5.8	2.921	0	0	3.0%	5.80 [0.07, 11.53]
Weerakiet	25.9	3.359	0	0	3.0%	25.90 [19.32, 32.48]
**Total (95% CI)**			**0**	**0**	**100.0%**	**33.09 [18.82, 47.35]**

Heterogeneity: $Tau^2 = 1729.82$; $Chi^2 = 11347.25$, df = 32 ($P < 0.00001$); $I^2 = 100\%$
Test for overall effect: Z = 4.55 ($P < 0.00001$)

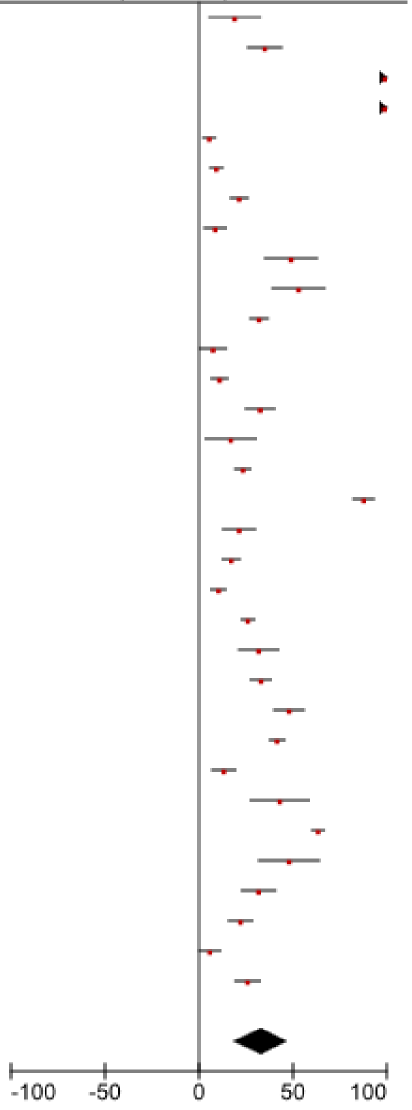

Figure S4. Forest plot of the prevalence of High TG among women with polycystic ovary syndrome.

Study or Subgroup	Prevalence	SE	Total	Total	Weight	Prevalence IV, Random, 95% CI
Alvarez-Blasco	72	7.937	0	0	3.0%	72.00 [56.44, 87.56]
Apridonidze	68	4.53	0	0	3.2%	68.00 [59.12, 76.88]
Bhattacharya	98.9	0.741	0	0	3.3%	98.90 [97.45, 100.35]
Bhattacharya(b)	98.7	0.805	0	0	3.3%	98.70 [97.12, 100.28]
Caliskan(a)	61	3.615	0	0	3.3%	61.00 [53.91, 68.09]
Carmina(a)	45.1	2.963	0	0	3.3%	45.10 [39.29, 50.91]
Cheung	28.6	2.631	0	0	3.3%	28.60 [23.44, 33.76]
Costa(a)	52.2	5.265	0	0	3.2%	52.20 [41.88, 62.52]
Coviello(a)	84	5.237	0	0	3.2%	84.00 [73.74, 94.26]
Ehrmann	66	2.469	0	0	3.3%	66.00 [61.16, 70.84]
Fruzzetti	43.4	6.807	0	0	3.1%	43.40 [30.06, 56.74]
Gambineri(a)	58	3.489	0	0	3.3%	58.00 [51.16, 64.84]
Glueck	64.5	4.073	0	0	3.2%	64.50 [56.52, 72.48]
Gulcelik	48	9.121	0	0	2.9%	48.00 [30.12, 65.88]
Hahn	44.8	2.452	0	0	3.3%	44.80 [39.99, 49.61]
Hosseinpanah	95.2	1.833	0	0	3.3%	95.20 [91.61, 98.79]
Indhavivadhana(a)	39.6	3.093	0	0	3.3%	39.60 [33.54, 45.66]
Kyrkou 2015	26.1	2.895	0	0	3.3%	26.10 [20.43, 31.77]
Madani	71.5	1.807	0	0	3.3%	71.50 [67.96, 75.04]
Mandrelle	91.7	2.518	0	0	3.3%	91.70 [86.76, 96.64]
Marcondes	67.6	5.477	0	0	3.2%	67.60 [56.87, 78.33]
Moini	68.8	2.758	0	0	3.3%	68.80 [63.39, 74.21]
Moradi	71	3.692	0	0	3.3%	71.00 [63.76, 78.24]
Ni	41.6	2.05	0	0	3.3%	41.60 [37.58, 45.62]
Park	45.1	4.68	0	0	3.2%	45.10 [35.93, 54.27]
Rabelo-Acevedo	71	7.266	0	0	3.0%	71.00 [56.76, 85.24]
Rong	85.9	1.205	0	0	3.3%	85.90 [83.54, 88.26]
Soares	69.6	4.554	0	0	3.2%	69.60 [60.67, 78.53]
Verit	42.3	3.869	0	0	3.2%	42.30 [34.72, 49.88]
Vrbikova	34.8	5.954	0	0	3.1%	34.80 [23.13, 46.47]
Weerakiet	59.4	3.766	0	0	3.2%	59.40 [52.02, 66.78]
**Total (95% CI)**			**0**	**0**	**100.0%**	**61.87 [53.31, 70.43]**

Heterogeneity: Tau² = 573.45; Chi² = 3353.20, df = 30 (P < 0.00001); I² = 99%

Test for overall effect: Z = 14.16 (P < 0.00001)

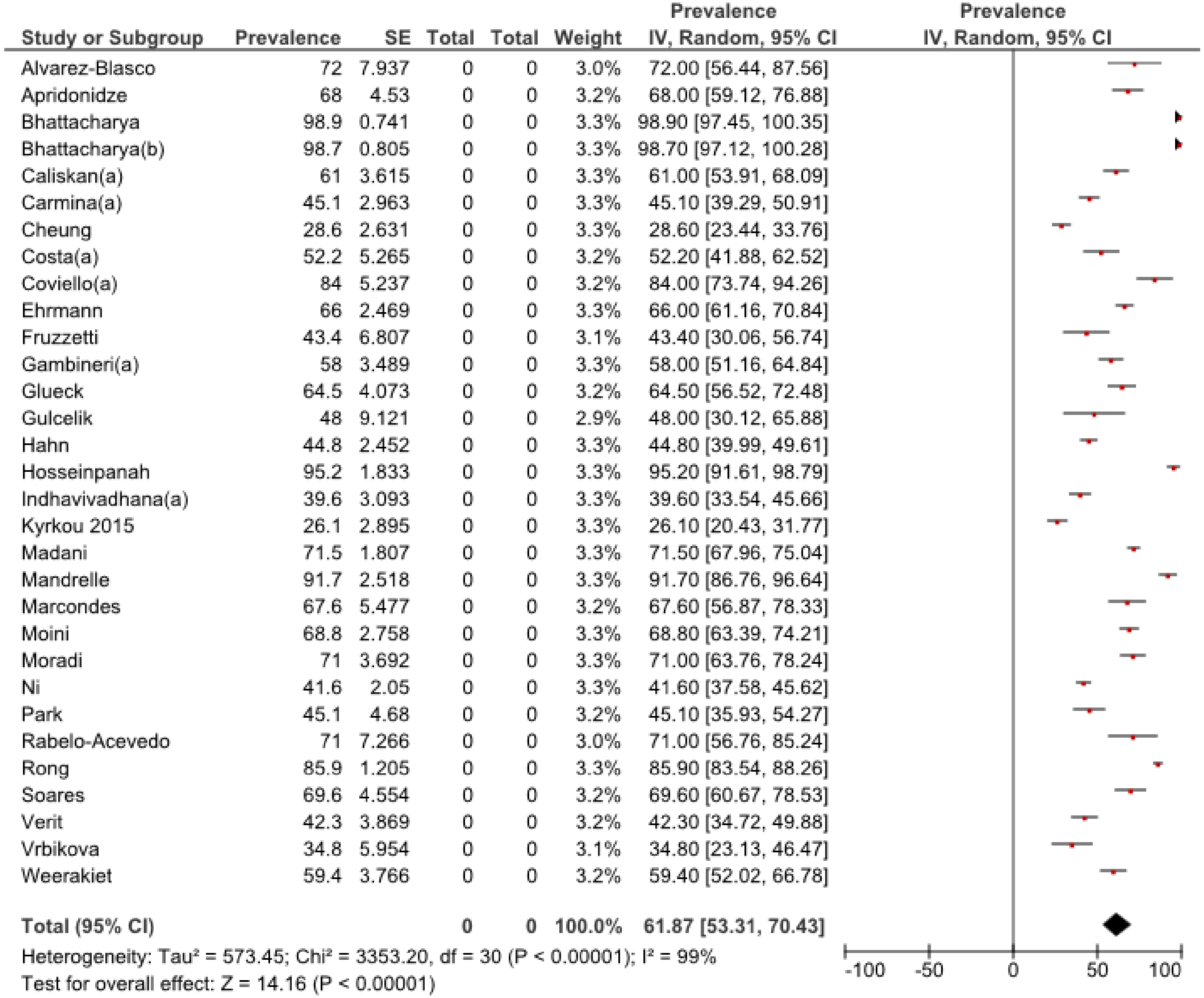

Figure S5. Forest plot of the prevalence of Low HDL among women with polycystic ovary syndrome.

Table S1. The risk of bias assessment for cross-sectional studies included into the meta-analysis

	1.Representativeness of the sample:	2.Sample size:	3.Non-respondents:	4.Ascertainment of the exposure (risk factor):	5. Confounding factors are controlled.	6.Assessment of the outcome:	7.Statistical test:	Overall Score
Pillai BP(2015)	C	b	c	B	-	c	a	3
Madani T(2015)	C	b	c	b	-	c	a	3
Shabir I(2014)	b	b	c	b	-	c	a	4
Figurova J(2014)	c	b	c	b	-	c	a	3
Kim MJ(2014)	b	b	c	b	a	c	a	5
Mandrelle K(2012)	c	a	c	b	a	c	a	5
Moini A(2012)	c	a	c	b	-	c	a	4
Ishak A(2012)	c	a	c	b	a	c	a	5
Bhattacharya SM(2011)	c	a	c	b	a	c	a	5
Mehrabian F(2011)	c	b	c	b	a	c	a	4
Gangale MF(2011)	c	b	c	b	-	c	a	3
Dey R(2011)	c	b	c	b	-	c	a	3

Bhattacharya SM(2010)	**c**	**b**	**c**	**b**	**-**	**c**	b	**3**
Indhavivadhana S(2010)	**b**	**a**	**c**	**b**	**-**	**c**	a	**5**
Fruzzetti F(2009)	**c**	**b**	**c**	**b**	**-**	**c**	a	**3**
Moradi S(2009)	**c**	**b**	**c**	**b**	**-**	**c**	a	**3**
Ni R(2009)	**c**	**b**	**c**	**b**	**-**	**c**	a	**3**
Soares EMM(2008)	**c**	**b**	**c**	**b**	**-**	**c**	a	**3**
Cheung LP(2008)	**c**	**a**	**c**	**b**	**a**	**c**	a	**5**
Weerakiet S(2007)	**c**	**b**	**c**	**b**	**-**	**c**	a	**3**
Marcondes JAM(2007)	**c**	**b**	**c**	**b**	**-**	**c**	a	**3**
Ehrmann DA(2006)	**b**	**b**	**c**	**b**	**-**	**c**	a	**4**
Coviello AD(2006)	**b**	**b**	**c**	**b**	**-**	**c**	a	**4**
Leibel NL (2006)	**b**	**b**	**c**	**b**	**-**	**c**	a	**4**
Apridonidze T(2005)	**b**	**b**	**c**	**b**	**b**	**c**	a	**5**
Rabelo-Acevedo M(2005)	**b**	**b**	**c**	**c**	**-**	**c**	b	**4**
Floria E(2004)	**b**	**b**	**c**	**b**	**-**	**c**	a	**4**

****indicating 2 scores**

*** indicating 1 score.**

- Minimum and maximum scores are 0 and 9, respectively.

1)

a) Truly representative of the average in the target population. * (all subjects or random sampling)
b) Somewhat representative of the average in the target population. * (non-random sampling)
c) Selected group of users.
d) No description of the sampling strategy.

2)

a) Justified and satisfactory. *
b) Not justified.

3)

a) Comparability between respondents and non-respondents characteristics is established, and the response rate is satisfactory. *
b) The response rate is unsatisfactory, or the comparability between respondents and non-respondents is unsatisfactory.
c) No description of the response rate or the characteristics of the responders and the non-responders.

4)

a) Validated measurement tool. **
b) Non-validated measurement tool, but the tool is available or described.*
c) No description of the measurement tool.

5)

a) The study controls for the most important factor (select one). *
b) The study control for any additional factor. *
